# Off-Label Use of Bedaquiline in Children and Adolescents with Multidrug-Resistant Tuberculosis

**DOI:** 10.3201/eid2310.170303

**Published:** 2017-10

**Authors:** Jay Achar, Cathy Hewison, Ana P. Cavalheiro, Alena Skrahina, Junia Cajazeiro, Parpieva Nargiza, Krzysztof Herboczek, Assliddin S. Rajabov, Jennifer Hughes, Gabriella Ferlazzo, James A. Seddon, Philipp du Cros

**Affiliations:** Médecins Sans Frontières, London, UK (J. Achar, K. Herboczek, J.A. Seddon, P. du Cros);; Médecins Sans Frontières, Paris, France (C. Hewison); Médecins Sans Frontières, Dushanbe, Tajikistan (A.P. Cavalheiro);; Republican Scientific and Practical Centre for Pulmonology and Tuberculosis, Minsk, Belarus (A. Skrahina);; Médecins Sans Frontières, Nukus, Karakalpakstan, Uzbekistan (J. Cajazeiro);; Ministry of Health, Tashkent, Uzbekistan (P. Nargiza);; State Enterprise of the Republican Center for the Protection of the Population from Tuberculosis, Dushanbe (A.S. Rajabov);; Médecins Sans Frontières, Cape Town, South Africa (J. Hughes, G. Ferlazzo);; Imperial College London, London (J.A. Seddon)

**Keywords:** MDR TB, XDR TB, pediatric, treatment, bedaquiline, tuberculosis, multidrug resistance, bacteria, antimicrobial resistance, respiratory infections, off-label use, United Kingdom, France, Tajikistan, Belarus, Uzbekistan, *Mycobacterium tuberculosis*, multidrug-resistant tuberculosis, children, adolescents, TB, South Africa

## Abstract

We describe 27 children and adolescents <18 years of age who received bedaquiline during treatment for multidrug-resistant tuberculosis. We report good treatment responses and no cessation attributable to adverse effects. Bedaquiline could be considered for use with this age group for multidrug-resistant tuberculosis when treatment options are limited.

The World Health Organization (WHO) estimated that nearly half a million persons became infected with multidrug-resistant (MDR) tuberculosis (TB), defined as disease caused by *Mycobacterium tuberculosis* strains resistant to rifampin and isoniazid, in 2015 (*1*). Modeling studies suggest that ≈32,000 of these cases occurred in children <15 years of age (*2*). Although limited information is available on the burden of extremely drug-resistant (XDR) TB (MDR TB with additional resistance to a fluoroquinolone and a second-line injectable drug) among children, >33% of children with MDR TB are estimated to exhibit additional resistance to fluoroquinolone, a second-line injectable drug, or both (*3*). Once a child with MDR TB is given the correct diagnosis and started on therapy, treatment outcomes are good (*4*). However, multiple challenges exist for children and adolescents with this disease. First, poor access to effective regimens and difficulties in establishing laboratory diagnoses continue to lead to inappropriate management of disease among many children. Second, adverse effects from MDR TB treatments are common; in 1 cohort, >25% of children receiving an injectable drug suffered hearing loss (*5*). Third, for children and adolescents infected with more extensively resistant strains, treatment options are limited.

In 2013, following US Food and Drug Administration approval of bedaquiline (in 2012), the WHO released interim guidance on the use of this drug (*6*). Key determinants of eligibility to receive bedaquiline included the inability to construct an effective 4-drug regimen using other available drugs or diagnosis with disease caused by strains with fluoroquinolone resistance. Limited available data to inform the use of bedaquiline in children led to the WHO stating that “Use of the drug in pregnant women and children is not advised due to a lack of evidence on safety and efficacy.” 

One large retrospective cohort analysis reported that up to two thirds of all patients with MDR TB might benefit from adding bedaquiline or delamanid to their treatment regimen (*7*). However, despite the US Centers for Disease Control and Prevention stating that bedaquiline use can be considered for children and adolescents when treatment options are limited, further studies to evaluate the drug in these groups have been slow to materialize. The bedaquiline compassionate use program conducted by the drug’s manufacturer, Janssen Pharmaceutical (Beerse, Belgium), excluded all patients <18 years of age (*8*). A Janssen-sponsored study (ongoing as of July 2017) will evaluate the antimycobacterial activity, pharmacokinetic profile, tolerability, and safety of bedaquiline among children and adolescents <18 years of age in South Africa, the Philippines, and Russia, with further sites planned in India (ClinicalTrials.gov nos. NCT02354014). Despite WHO guidance to avoid using bedaquiline in patients <18 years of age (*6*), some clinicians have done so when options were limited. The aim of this report was to describe experiences treating children and adolescents with MDR TB with drug regimens that included bedaquiline.

## The Study

We collected data on patients <18 years of age from the TB treatment programs supported by Médecins Sans Frontières in South Africa, Tajikistan, and Uzbekistan and the National TB Programme in Belarus. During November 2014–January 2017, a total of 27 children and adolescents began regimens containing bedaquiline (Table). Median age was 16 (range 10–17) years, and 15 (56%) were girls. Median weight was 50 (range 35–76) kg. No patients were HIV positive. One male patient had intrathoracic lymph node TB, and 26 patients had pulmonary TB. Diagnoses for 17 (63%) patients were confirmed by mycobacterial culture. Baseline sputum smears from 19 (70%) patients were positive for acid-fast bacilli. One boy had concomitant spinal TB osteomyelitis. Most patients (18/27, 67%) had XDR TB; 6 (22%) had MDR TB with fluoroquinolone resistance; and 3 (11%) had MDR TB with resistance to a second-line injectable drug. For the 10 patients without positive mycobacterial cultures, drug susceptibility was presumed from contact history. Thus, for all patients, the decision to use bedaquiline was based on confirmed or presumed extensive drug resistance that resulted in the inability to construct an effective treatment regimen.

**Table:** Demographic, treatment, and outcome characteristics of a cohort of 27 children <18 years of age receiving bedaquiline for the treatment of MDR TB*

Characteristic	No. (%)
Country	
Belarus	15 (56)
South Africa	3 (11)
Tajikistan	6 (22)
Uzbekistan	3 (11)
Age, y, median (range)	16 (10–17)
Sex	
Female	15 (56)
Male	12 (44)
Weight, kg, median (range)	50 (35–76)
Body mass index, kg/m^2^, median (IQR)	18.5 (17.2–19.6)
Cavities on baseline chest radiograph, n = 24	9 (38)
Baseline sputum smear positive	19 (70)
Baseline sputum culture positive	17 (63)
Baseline drug resistance pattern	
MDR TB	0 (0)
Pre–XDR TB	
Resistant to second-line injectable	3 (11)
Resistant to fluoroquinolone	6 (22)
XDR TB	18 (67)
Resistant drugs,† median (IQR), n = 24	5 (5–6)
Drugs in initial treatment regimen, median (IQR)	6 (6–7)
Drugs included in treatment regimen	
Moxifloxacin	6 (22)
Clofazimine	26 (96)
Linezolid	26 (96)
Imipenem	4 (15)
Bedaquiline treatment duration if completed, d, median (IQR), n = 20	172 (168–178)
Sputum culture negative at February 24, 2017, n = 23	23 (100)
Sputum culture negative after 24 wks of bedaquiline, n = 22‡	22 (100)
Reported adverse effects	
No grade 3 or 4	19 (70)
Grade 3 or 4, not caused by bedaquiline	3 (11)
Grade 3 or 4, caused by bedaquiline	5 (19)§

*Values are no. (%) patients except as indicated. IQR, interquartile range; MDR TB, multidrug-resistant tuberculosis; XDR TB, extensively drug-resistant tuberculosis; QTcF, QT interval corrected using the Fridericia formula. †Resistance among the following drugs were considered: isoniazid, rifampin, ethambutol, pyrazinamide, kanamycin, any fluoroquinolone, amikacin, and capreomycin. ‡Twenty-seven children completed 24 weeks of bedaquiline, but data were available for 22. §All 5 were children who experienced prolongation of QTcF.

The high proportion of patients with resistance to second-line drugs led to frequent use of repurposed drugs, such as linezolid (26/27, 96%) and clofazimine (26/27, 96%). Intravenous imipenem was used in some patients (4/27, 15%). Despite concerns about potential additive cardiac toxicity when combining bedaquiline and moxifloxacin, combined treatment was judged necessary for 6 (22%) children. Five of these 6 children also received clofazimine in their regimen. All cases except 1 received the recommended adult dosing regimen for bedaquiline (400 mg/d for 2 weeks, then 200 mg 3×/wk for 6 months). One 10-year-old girl (weighing 35 kg) received 300 mg/d (recommended on the basis of expert opinion) during her loading phase.

The mean duration of bedaquiline treatment of the 20 children and adolescents who completed therapy was 172 days. Regular (weekly for most) electrocardiogram monitoring was performed for all cases. Fridericia’s formula was used to correct the measured QT intervals (QTcF) for the heart rate, and cardiotoxicity was defined according to the National Cancer Institute Common Terminology Criteria for Adverse Events version 4.03 (https://evs.nci.nih.gov/ftp1/CTCAE/CTCAE_4.03_2010-06-14_QuickReference_8.5x11.pdf). Five patients had grade 3 or 4 prolongation: 2 received bedaquiline, clofazimine, and moxifloxacin and 3 received bedaquiline and clofazimine. Four patients experienced increases in QTcF >60 ms above baseline: 2 during the first month of treatment, 1 after 3 months of treatment, and 1 after 6 months of treatment. All 4 patients improved <1 month following electrolyte replacement, and no drug cessation was required. Recurrent prolongation of QTcF >500 ms was identified in 1 adolescent during the first 6 months of treatment and necessitated cessation of moxifloxacin and clofazimine after electrolyte replacement showed insufficient benefit. Following stoppage of these drugs, QTcF returned to normal. No patient experienced symptoms attributable to prolongation of QTcF during treatment with bedaquiline.

As of February 24, 2017, of the 23 patients who remained on treatment and had data available, all were culture negative; 14 of these 23 had been positive at baseline. No clinical signs suggestive of treatment failure were noted among patients of this cohort.

## Conclusions

Our experience suggests that bedaquiline can be used safely in children >12 years of age with appropriate monitoring and could be considered in younger children in select circumstances when benefits are likely to outweigh risks. Although treatment outcomes are preliminary, we report good responses to treatment with bedaquiline among a group of children and adolescents with advanced resistance to second-line drugs. Although prolongation of QTcF was noted in some (5/27) patients when concomitant cardiotoxic drugs were used, no patient required bedaquiline cessation.

Continued reluctance to use contact history for diagnosing advanced drug resistance and limited availability of drug susceptibility testing in children remain barriers for the consideration of new drugs and use of appropriate MDR TB regimens. In addition, restricted availability of delamanid in TB programs and the perceived age restriction on the use of bedaquiline has resulted in children failing to benefit from drugs that are being used safely and successfully in adults. Although the lack of pharmacokinetic data on bedaquiline in children and adolescents must be addressed, other second-line TB drugs have been recommended and prescribed despite insufficient data on pharmacokinetics. Expanding access to bedaquiline and delamanid for children could lead to the reduction in the need for second-line injectable drugs, which are strongly associated with irreversible toxicity (*5*). This experience supports similar recommendations given by the US Centers for Disease Control and Prevention (*9*) and an international group of pediatric TB experts (*10*).
